# Left Atrial Wall Motion Velocity Assessed during Atrial Fibrillation Predicts Sinus Rhythm Maintenance after Electrical Cardioversion in Patients with Persistent Atrial Fibrillation

**DOI:** 10.3390/ijerph192315508

**Published:** 2022-11-23

**Authors:** Paweł Wałek, Joanna Roskal-Wałek, Patryk Dłubis, Justyna Tracz, Beata Wożakowska-Kapłon

**Affiliations:** 1 Collegium Medicum, Jan Kochanowski University, 25-317 Kielce, Poland; 2 1st Clinic of Cardiology and Electrotherapy, Swietokrzyskie Cardiology Centre, 25-736 Kielce, Poland; 3 Ophthalmology Clinic, Voivodeship Regional Hospital, 25-736 Kielce, Poland; 4 Clinic of Neurology, Swietokrzyskie Neurology Center, 25-736 Kielce, Poland

**Keywords:** atrial fibrillation, cardioversion, tissue Doppler, remodeling, echocardiography

## Abstract

Reduced left atrial wall motion velocity measured during AF (LAWMV) indicates left atrial remodeling. The aim of this study was to investigate whether LAWMV assessed with tissue Doppler imaging during atrial fibrillation (AF) predicts sinus rhythm (SR) maintenance after direct current cardioversion (DCCV) for persistent AF. The study included 126 patients who underwent DCCV and were followed for 12 months. At 12 months, maintained SR was reported in 55 patients (43.7%). We noted that LAWMV was higher in patients with maintained SR at 12 months than in those with recurrent AF (3.69 ± 0.84 vs. 2.86 ± 1.09; *p* < 0.001). In the multivariable regression model containing echocardiographic variables, LAWMV was an independent predictor of SR maintenance at 12 months (odds ratio [OR] 1.72, 95% confidence interval [CI] 1.1–2.69; *p* = 0.017). Similarly, LAWMW was an independent predictor of SR maintenance at 12 months (OR 1.81, 95% CI 1.19–2.77; *p* = 0.006) in the multivariate regression model containing both echocardiographic and clinical variables. LAWMV predicts SR maintenance after DCCV for persistent AF. Echocardiographic markers of left atrial mechanical remodeling are better at predicting SR maintenance than markers of structural remodeling.

## 1. Introduction

Atrial fibrillation (AF) is a type of arrhythmia characterized by rapid and uncoordinated electrical and mechanical activation of the atria [1,2]. It is the most common supraventricular arrhythmia, and its incidence is expected to rise in the coming years. Based on duration and frequency, AF can be divided into paroxysmal, persistent, and permanent [1,2,3].

So far, numerous studies have been published that assessed the clinical, biochemical, and echocardiographic risk factors for recurrent AF after direct current cardioversion (DCCV) [4,5,6,7,8]. The remodeling of the atrial wall is one of the key pathological mechanisms involved in the development of AF.

Atrial remodeling may be caused by AF, but it can also be one of the factors that contribute to subsequent AF episodes [9]. It is classified into electrical, mechanical, and structural remodeling. There is a link between the severity of atrial remodeling and the onset of subsequent AF episodes as well as the progression from paroxysmal to persistent AF [1]. By assessing the stage of atrial remodeling, it is possible to predict the efficacy of DCCV in patients with persistent AF [10,11,12,13,14,15,16,17,18,19,20,21,22,23,24,25,26,27,28,29,30,31,32,33].

To date, numerous echocardiographic parameters that allow the prediction of sinus rhythm (SR) maintenance after DCCV have been described, including parameters of structural, mechanical, and electrical remodeling, as well as of left ventricular filling pressure. Some of them can be assessed using standard transthoracic echocardiography, whereas others require advanced techniques such as strain and strain rate measurements and transesophageal echocardiography. The more advanced techniques offer better possibilities for assessing SR maintenance than standard echocardiographic measures such as left atrial dimension or volume. However, the use of advanced measurements or transesophageal echocardiography is time consuming and requires additional software and echocardiographic probes. Moreover, some of the above parameters can be assessed during SR (i.e., after successful DCCV), whereas it would be optimal to predict DCCV efficacy before the procedure (i.e., during AF).

In this study, we assessed the prognostic value of LAWMV measured using tissue Doppler imaging during AF in patients referred for DCCV due to persistent AF.

## 2. Materials and Methods

### 2.1. Study Population

Informed consent to participate in the study was obtained from each patient. The study protocol was approved by the Institutional Review Board of the Świętokrzyskie Medical Chamber. Patients presenting with persistent AF who underwent DCCV between February 2017 and August 2020 were included in the study. The inclusion criteria were as follows: symptomatic persistent AF lasting at least 7 days, ejection fraction during SR ≥ 40%, and adequate anticoagulation for at least 3 weeks before cardioversion (warfarin, dabigatran, acenocoumarol, apixaban, or rivaroxaban). The exclusion criteria were age < 18 years, lack of consent to participate in the study or to undergo cardioversion, failed acquisition of left atrial wall velocity, poor-quality echocardiographic images, valvular prosthesis, moderate or severe valve regurgitation or stenosis, the presence of left atrial appendage thrombus, acute myocardial infarction, acute decompensation of heart failure, previous pulmonary vein isolation, dysthyroidism, anemia with hemoglobin levels < 6.9 mmol/L, immune disease, acute infection, and neoplastic disease.

Clinical and echocardiographic data as well as blood samples were collected directly before cardioversion. Patients were followed clinically and with electrocardiography at 1, 6, and 12 months. In addition, all patients with SR underwent 24-h ambulatory Holter monitoring at 1 and 12 months. Patients were asked to report to our Cardiology Department when they experienced palpitations or when routine medical care revealed the recurrence of AF.

### 2.2. Clinical Data

The following clinical data were recorded at baseline: age, sex, body mass index, diabetes mellitus, hypertension, smoking status, history of stroke or transient ischemic attack, coronary artery disease, heart failure, and medical treatment. Patients were diagnosed with coronary artery disease if they had a history of myocardial infarction, percutaneous coronary intervention, or coronary artery bypass grafting. The glomerular filtration rate was measured using the Modification of Diet in Renal Disease formula. The CHA_2_DS_2_-VASc score was assessed according to the European guidelines [1] on the management of AF.

### 2.3. Sinus Rhythm Restoration

General anesthesia was used for all DCCV procedures. If the effectiveness of anticoagulation was uncertain, transesophageal echocardiography was performed to assess for left atrial thrombi. All DCCVs were performed with paddles placed in the anterolateral position, and a biphasic defibrillator was used to deliver an electrical shock at 150 J to 300 J. If this proved ineffective, another shock was administered with an additional 100 J. Successful cardioversion was defined as SR maintenance for ≥24 h after the procedure. Patients with SR received anticoagulation, upstream therapy, or antiarrhythmic drugs according to clinical judgment. Based on the risk of AF recurrence, antiarrhythmic drugs (propafenone or amiodarone) were prescribed by a physician who was blinded to the echocardiographic data assessed in this study.

### 2.4. Echocardiographic Evaluation

Transthoracic echocardiography was performed based on current guidelines [34,35] using the Vivid S6 Echocardiographic device (General Electric Medical Systems, Horten, Norway) equipped with an M4S RS probe. All examinations were done by a single investigator before DCCV. Standard M-mode Doppler imaging and 2-dimensional cine loops of the parasternal long- and short-axis views as well as apical 2-, 3-, and 4-chamber views were obtained for each participant. All echocardiographic images and measurements were obtained from standard views and stored digitally. The images were then retrieved and analyzed using offline software (EchoPAC PC Software, GE Medical Systems, Chicago, IL, USA). The maximum end-systolic left atrial volume (LAV) and the minimum end-diastolic left atrial volume (LAEDV) were assessed using the Simpson’s method from the apical 4- and 2-chambers views. The maximum LAV was measured at end-systole, on the frame just before mitral valve opening, by tracing the inner border of the atrium and taking care to avoid the area under the valve annulus, appendage, and pulmonary veins. The LAV was indexed to body surface area (LAVI). The minimum LAEDV was measured at the end of ventricular diastole, on the frame of mitral valve closure, and indexed to body surface area (LAEDVI). Left ventricular ejection fraction (LVEF) and left ventricular volume were assessed using the Simpson’s method. The right atrial area was measured at end-systole and end-diastole from the apical 4-chamber view, on the frame with tricuspid valve closure. Blood flow velocities were assessed by transmitral pulsed-wave Doppler imaging from the apical 4-chamber view with a sample volume of 2 mm placed between the tips of the mitral leaflets. Tissue Doppler imaging of the mitral annular motion was performed from the apical 4-chamber view with a sample volume of 5 mm at the lateral and septal basal regions. The s′ mean and e′ mean values were calculated as averages from septal and lateral measurements. The measurements obtained during AF were calculated by averaging data from 5 consecutive beats.

**Figure 1:**
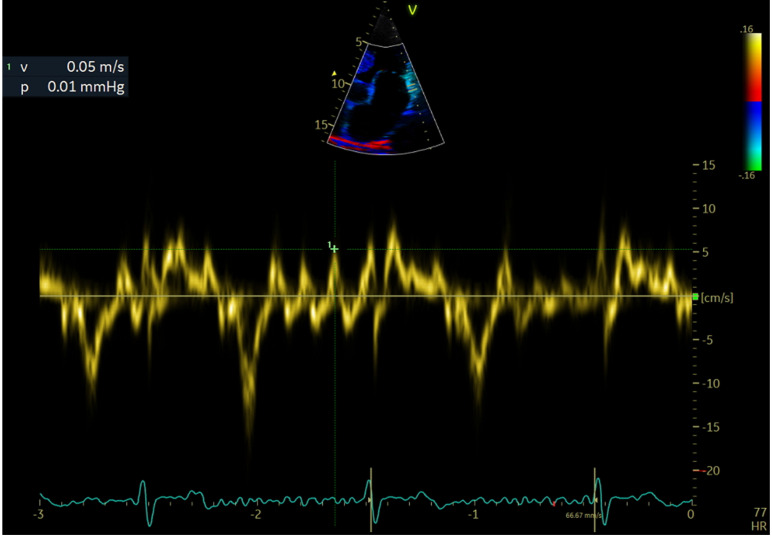
Left atrial wall motion velocity measurement. LAWMV of 5 cm/s towards the probe.

**Figure 2:**
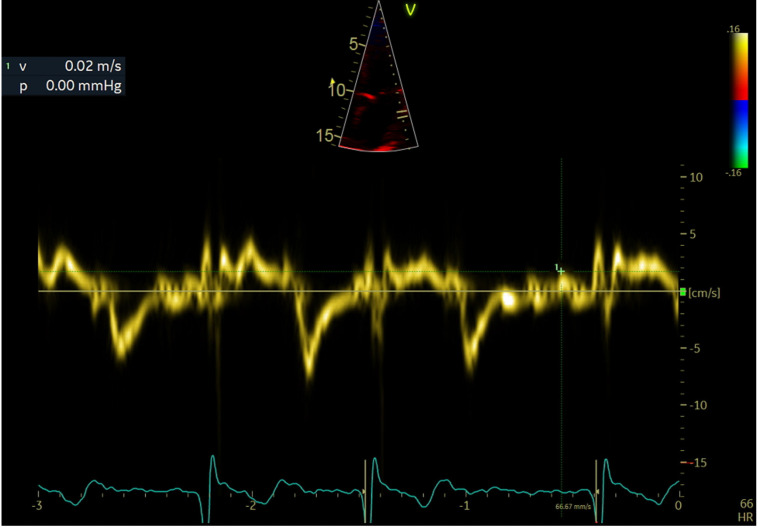
Left atrial wall motion velocity measurement. LAWMV of 2 cm/s towards the probe.

**Figure 3:**
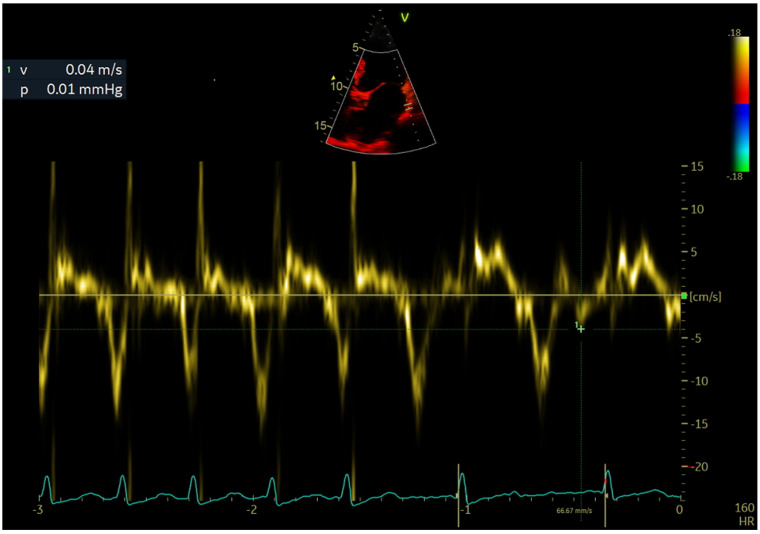
Left atrial wall motion velocity measurement during rapid atrial activity. Left atrial wall motion velocity measurement during a short pause in atrial activity. LAWMV of 4 cm/s from the probe.

LAWMV was measured directly using transthoracic echocardiography with tissue Doppler imaging in the apical 4-chamber view with a 4-mm sample volume. The Doppler gate was placed approximately 10 mm below the lateral mitral annulus (Figure 1, Figure 2 and Figure 3).

Velocity measurements were taken for about 3 s. The view angle was obtained by positioning the lateral left atrial wall as much as possible along the direction of the ultrasound wave propagation.

LAWMV was measured based on AF waves recorded just before the QRS complexes, after the wave corresponding to the e′ wave of the diastolic mitral annular motion. The measurements of the highest LAWMV towards the probe or from the probe were analyzed. The highest LAWMV value was selected for analysis. Considering that the s′ wave might coincide with the systolic mitral annular motion, no measurements were taken during the QRS complex. In addition, LAWMV was not measured in patients with AF with rapid ventricular activity in whom the cardiac pause was not recorded during echocardiography.

### 2.5. Statistical Analysis

Results were described as means ± standard deviations. Categorical variables were presented as counts and percentages. Normally distributed variables were compared using the Student *t*-test, and non-normally distributed variables were compared with the Mann—Whitney test or the chi-squared test.

Clinical and echocardiographic parameters with the lowest *p* values, as determined in the univariate logistic analysis, were included in a multivariable logistic analysis. Two multivariate models were created, one including selected clinical and echocardiographic variables with the lowest *p* value and the other including only echocardiographic variables with the lowest *p* value. The selected echocardiographic variables were parameters of structural and mechanical remodeling as well as of left ventricular filling pressure.

The receiver operating characteristic curves for predicting SR restoration and maintenance at 1, 6, and 12 months were calculated for LAWMV. The areas under the curve (AUC) and optimal cut-offs based on Youden’s J statistic were also calculated.

Statistical significance was set at *p* < 0.05. Statistical analyses were performed with STATISTICA 13.3 software (TIBCO Software Inc., Tulsa, OK, USA).

## 3. Results

Of the 126 patients included in the study, SR restoration after electrical cardioversion was noted in 107 (84.9%). At 12 months, 55 patients (43.7%) showed maintained SR. Patients who underwent successful DCCV and maintained SR at 12 months more often were male, had higher glomerular filtration rate, more often used beta-blockers before DCCV, and less often used diuretics before and after DCCV, as compared with patients without maintained SR at 12 months. Detailed clinical characteristics are presented in Table 1.

Patients who underwent successful DCCV and maintained SR at 12 months showed a greater size of the right ventricular outflow tract (RVOT), higher LVEF, lower LAV and LAVI, higher left atrial emptying fraction, higher systolic and early diastolic velocities on mitral annular tissue Doppler imaging, lower early filling wave, and higher LAWMV. Detailed echocardiographic data are presented in Table 2.

In the univariate regression analysis, the significant predictors of SR maintenance at 12 months after DCCV were male sex (odds ratio [OR] 2.74, 95% confidence interval [CI] 1.29–5.83; *p* = 0.009), glomerular filtration rate (OR 1.03, 95% CI 0.01–1.06; *p* = 0.01), diuretic use before (OR 0.41, 95% CI 0.2–0.85; *p* = 0.016) and after DCCV (OR 0.36, 95% CI 0.18–0.76; *p* = 0.007), greater size of the right ventricular outflow tract (OR 1.11, 95% CI 1.01–1.22; *p* = 0.032), LVEF (OR 1.02, 95% CI 1.001–1.04; *p* = 0.041), LAVI (OR 0.94, 95% CI 0.91–0.98; *p* < 0.001), left atrial emptying fraction (OR 1.09, 95% CI 1.05–1.15; *p* < 0.001), systolic mitral annular velocity (OR 1.65, 95% CI 1.25–2.17; *p* < 0.001), early diastolic mitral annular velocity (OR 1.36, 95% CI 1.13–1.63; *p* = 0.001), early filling wave (OR 0.06, 95% CI 0.01–0.43; *p* = 0.006), and LAWMV (OR 2.29, 95% CI 1.54–3.39; *p* < 0.001).

In the multivariable logistic regression analysis including echocardiographic and clinical variables, only LAWMV was a significant predictor of SR maintenance at 12 months (Table 3).

Similarly, in the multivariable logistic regression analysis including only echocardiographic variables with the lowest *p* value, only LAWMV was a significant predictor of SR maintenance at 12 months (Table 4).

**Figure 4:**
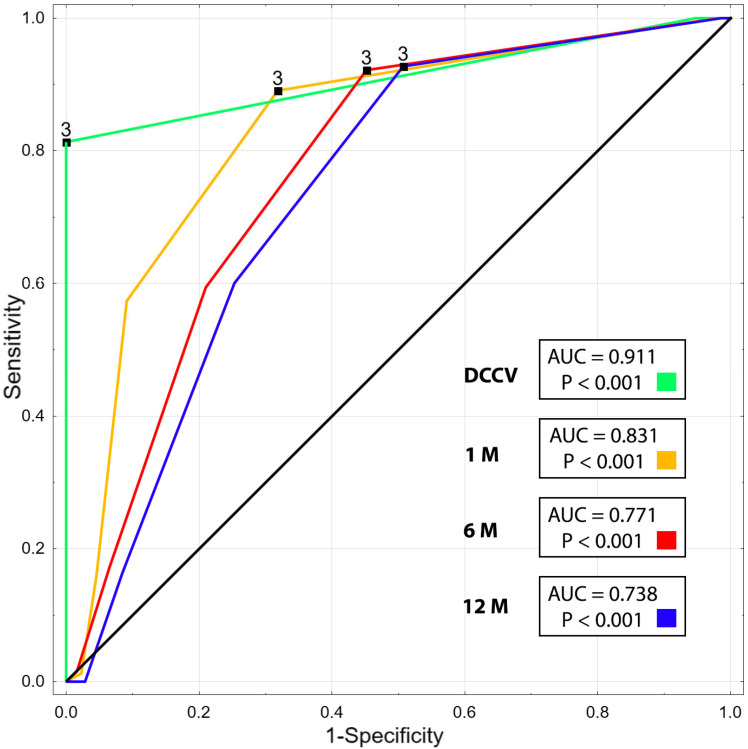
Receiver operating characteristic curve analysis of left atrial wall motion velocity for predicting the efficacy of direct current cardioversion and sinus rhythm maintenance at 1, 6, and 12 months.

In the receiver operating characteristic curve analysis, LAWMV was a predictor of SR restoration following DCCV (AUC = 0.911; *p* < 0.001) as well as SR maintenance at 30 days (AUC = 0.831; *p* < 0.001), 6 months (AUC = 0.771; *p* < 0.001), and 12 months (AUC = 0.738; *p* < 0.001) after DCCV (Figure 4).

The optimal cutoff values of LAWMV with sensitivity, specificity, negative and positive predictive values for predicting sinus rhythm restoration after DCCV, and sinus rhythm maintenance at 12 months after DCCV are presented in Table 5 and Table 6, respectively.

## 4. Discussion

The current study showed that LAWMV predicts the immediate success of DCCV in restoring and maintaining SR in patients with persistent AF. The highest prognostic value was noted for immediate successfully DCCV, with a subsequent decrease along with follow-up duration. The study showed that echocardiographic parameters of left ventricular function (left ventricular stroke volume, s′ wave), left ventricular filling pressure (E and e′ waves), structural remodeling (LAVI, LAEDVI), and mechanical remodeling have a prognostic value in terms of SR maintenance after DCCV in patients with persistent AF. However, in the multivariate regression models, only LAWMV (an echocardiographic marker of mechanical remodeling) was an independent predictor of SR maintenance after DCCV.

Previous studies assessed LAWMV in terms of its prognostic value for predicting SR maintenance after electrical cardioversion, but the measurements were done from color tissue Doppler images using the Q-Analysis software, and not directly as in our study [11]. The assessment of LAWMV using Q-Analysis requires additional software and is time consuming. In addition, in Q-Analysis, the frequency of obtaining velocity measurements is affected by the size of the color Doppler recording. On the other hand, when using tissue Doppler imaging for direct LAWMV measurement, no extra time or software is required. In addition, the measurement is done in the same way as that of early (e′) and late (a′) diastolic mitral annular velocity, but the Doppler gate should be placed approximately 10 mm below the mitral annulus. Color Doppler imaging and Q-Analysis have the obvious advantage of retrospective measurements from the previous recordings.

Numerous studies confirmed the prognostic value of echocardiographic parameters describing structural remodeling for predicting successful DCCV and the risk of recurrent AF [12,13,14]. In recent years, numerous reports also indicated the prognostic value of echocardiographic parameters assessing mechanical atrial remodeling for predicting DCCV efficacy and SR maintenance after DCCV [11,16,17,18,19,21,22,23,24,25,26,27,28,30,31,32]. The parameters describing functional remodeling can be assessed both by transthoracic and transesophageal echocardiography. In addition, they can be assessed both during AF and during SR.

Transthoracic echocardiography allows the assessment of left atrial contractility by measuring the left atrial emptying fraction. It was shown that left atrial emptying fraction assessed after successful DCCV (i.e., during SR) as well as during AF before DCCV has a higher prognostic value for predicting SR maintenance as compared with markers of structural remodeling [17,31]. Kim et al. reported that the presence of left atrial fibrillatory contraction flow, measured with pulsed-wave Doppler technique immediately after early diastolic mitral inflow during AF, predicted long-term success of DCCV [24]. It was also shown that the assessment of left atrial deformation both by tissue Doppler imaging and by a more recent method, speckle-tracking echocardiography, allows a better prediction of SR maintenance after DCCV [21,22,23,25,26,27,28,30,32]. Currently, speckle-tracking echocardiography is preferred for the evaluation of the cardiac strain and strain rate because it is free from an error due to the angular relationship of the measurements. Left atrial strain measurements are also used to assess the prognosis of SR maintenance after ablation of pulmonary vein isolation due to AF [36].

Mechanical remodeling can be also assessed by measuring the contractility of the left atrial appendage. It was shown that the greater the contractility and left atrial appendage ejection fraction, the better the prediction of SR maintenance after DCCV [16,18,19]. In our previous study on left atrial appendage wall motion velocity, the markers of mechanical remodeling had a higher prognostic value for predicting successful DCCV and SR maintenance than markers of structural remodeling [19]. However, the measurement of left atrial appendage wall motion velocity requires transesophageal echocardiography, although it was reported that the measurement can also be done during transthoracic echocardiography [37]. The method of measuring LAWMV presented by our team allows for a non-invasive, quick, and easily accessible assessment of the chance of maintaining SR after DCCV is performed due to persistent AF. Transesophageal echocardiography requires preparation of the patient for this examination, qualified medical staff, echocardiographic equipment, and may expose the patient to complications from the oral cavity, pharynx, and esophagus. In our previous work describing the usefulness of LAAWMV measurement in predicting DCCV efficacy in patients with persistent AF, we used TEE to measure LAAWMV [19]. In our opinion, measurements of left atrial myocardial velocities can be performed with greater accuracy using TEE than TTE, but we did not perform a comparative analysis between LAWMV measurements in TTE and LAAWMV measurements in TEE. Let the comparative analyzes between these methods be an inspiration for further research on the use of these techniques in patients with persistent AF who are undergoing DCCV or qualified for ablation.

### Strengths and Limitations

Among the advantages, the LAWMV measurement is easy to perform and is not time consuming. It is done in the same way as the measurement of mitral annular motion velocity, but the Doppler gate should be placed approximately 10 mm below the annulus. In our study, LAWMV measurements were done using tissue Doppler imaging. The technique is dependent on the angle of the Doppler beam with respect to the moving object, which affects the outcome. The use of speckle-tracking echocardiography might eliminate a possible error resulting from the suboptimal placement of the ultrasound beam, but this technique requires additional software and may be time consuming. Measuring LAWMV requires a pause in the ventricular systolic function to visualize the left atrial wall contraction wave. When patients have a very fast ventricular rate, it is difficult to measure LAWMV (Figure 3).

The limitations of our study include a single-center design and a small number of patients. When interpreting our results, it is important to bear in mind that echocardiography is operator-dependent and requires skill and expertise. Therefore, all echocardiographic measurements in our study were taken by a single experienced investigator. The use of constant heart rhythm monitoring was not feasible in our long-term study; thus, it is possible that we missed self-limiting episodes of AF recurrence. All DCCV procedures were performed in the anterolateral position, without changing the paddle position in case of cardioversion failure, which may have influenced the success rate.

In this study, we did not assess AF duration because we were not able to determine it reliably. AF is often asymptomatic, or its symptoms can develop slowly. Many of our patients were unable to report the onset of AF.

The levels of brain natriuretic peptide and N-terminal pro-B-type natriuretic peptide were not measured in all patients, although it may have been an important variable for statistical analysis, considering the confirmed effect of diuretics.

## 5. Conclusions

The measurement of LAWMV during AF predicts the immediate success of DCCV and SR maintenance at 12 months after DCCV. The results of this study support the use of left atrial mechanical remodeling parameters over structural remodeling parameters to predict SR maintenance after DCCV. Considering the ongoing advances in ablation techniques, we encourage clinicians to study the echocardiographic markers of mechanical atrial remodeling for predicting SR maintenance after AF ablation as well as the risk of recurrent AF in patients after ablation for typical atrial flatter.

## Figures and Tables

**Table 1:** Clinical characteristics.

Variables	Study Population *n* = 126 (100%)	Sinus Rhythm Maintenance *n* = 55 (43.7%)	Cardioversion Failure or AF Recurrence *n* = 71 (56.7%)	*p*-Value
Age (years), mean (SD)	65.10 (10.17)	63.38 (11.64)	66.44 (8.74)	0.273
Age < 65 years, *n* (%)	49 (38.89)	24 (43.64)	25 (35.21)	0.486
Age 65–74 years, *n* (%)	58 (46.03)	22 (40.00)	36 (50.70)	
Age ≥ 75 years, *n* (%)	19 (15.08)	9 (15.08)	10 (14.08)	
Male sex, *n* (%)	75 (59.52)	40 (72.73)	35 (49.30)	0.008
BMI (kg/m^2^), mean (SD)	30.48 (4.64)	30.80 (3.99)	30.24 (5.11)	0.291
Hypertension, *n* (%)	107 (84.92)	45 (81.82)	62 (87.32)	0.392
Diabetes mellitus, *n* (%)	28 (22.22)	13 (23.64)	15 (21.13)	0.737
Coronary artery disease, *n* (%)	20 (15.87)	9 (16.36)	11 (15.49)	0.895
Stroke/TIA, *n* (%)	12 (9.52)	4 (7.27)	8 (11.27)	0.449
CHA_2_DS_2_-VAScscore, mean (SD)	2.83 (1.54)	2.65 (1.49)	2.97 (1.57)	0.243
CHA_2_DS_2_-VASc = 0, *n* (%)	6 (4.76)	3 (5.45)	3 (4.23)	0.863
CHA_2_DS_2_-VASc = 1, *n* (%)	21 (16.67)	10 (18.18)	11 (15.49)	
CHA_2_DS_2_-VASc ≥ 2, *n* (%)	99 (78.57)	42 (76.36)	57 (80.28)	
Smokers, *n* (%)	10 (7.94)	3 (5.45)	7 (9.86)	0.364
Heart failure, *n* (%)	40 (31.75)	20 (36.36)	20 (28.17)	0.327
MDRD (mL/m^2^) mean (SD)	64.79 (16.67)	69.28 (16.59)	61.31 (15.99)	0.005
Creatinine (mg/dL), mean (SD)	1.13 (0.23)	1.10 (0.19)	1.14 (0.25)	0.454
TnT (ng/L), *n* = 126, mean (SD)	11.23 (8.80)	11.86 (8.70)	10.75 (8.91)	0.685
HbA1c (%), *n* = 106, mean (SD)	6.11 (0.88)	6.02 (0.91)	6.17 (0.85)	0.156
Medication use before DCCV				
Beta-blockers, *n* (%)	114 (90.48)	53 (96.36)	61 (85.92)	0.048
Amiodarone, *n* (%)	13 (10.32)	4 (7.27)	9 (12.68)	0.322
ACE inhibitors/ARB, *n* (%)	106 (84.13)	46 (83.64)	60 (84.51)	0.894
Statins, *n* (%)	81 (64.29)	38 (69.09)	43 (60.56)	0.322
Diuretics, *n* (%)	59 (46.83)	19 (34.55)	40 (56.34)	0.015
Spironolactone/eplerenone, *n* (%)	23 (18.25)	13 (23.64)	10 (14.08)	0.169
Medication use post DCCV				
Beta-blockers, *n* (%)	104 (82.54)	48 (87.27)	56 (78.87)	0.218
Amiodarone, *n* (%)	40 (31.75)	17 (30.91)	23 (32.39)	0.859
Propafenone, *n* (%)	33 (26.19)	17 (30.91)	16 (22.54)	0.289
ACE inhibitors/ARB, *n* (%)	104)82.54)	46 (83.64)	58 (81.69)	0.775
Statins, *n* (%)	81 (64.29)	35 (63.64)	46 (64.79)	0.893
Diuretics, *n* (%)	61 (48.41)	19 (34.55)	42 (59.15)	0.006
Spironolactone/eplerenonen (%)	28 (22.22)	15 (27.27)	13 (18.31)	0.230

ACE inhibitors/ARB, angiotensin-converting enzyme inhibitors/angiotensin II receptor blockers; BMI, body mass index; HbA1c, glycated hemoglobin; MDRD, glomerular filtration rate; post, after cardioversion; pre, before cardioversion; SD, standard deviation; TIA, transient ischaemic attack; TnT, troponin T.

**Table 2:** Echocardiographic characteristics.

Variable	Study Population *n* = 126 (100%)	Sinus Rhythm Maintenance *n* = 55 (43.7%)	Cardioversion Failure or AF Recurrence *n* = 71 (56.7%)	*p*-Value
RVOT (mm)	31.17 (3.90)	32.04 (4.11)	30.51 (3.62)	0.029
IVS (mm)	10.73 (1.69)	10.69 (1.67)	10.76 (1.71)	0.926
LVEDD (mm)	51.13 (6.50)	51.62 (6.31)	50.75 (6.66)	0.457
LVESD (mm)	35.92 (7.60)	36.44 (7.73)	35.52 (7.52)	0.504
LVEDV (mL)	117.21 (34.60)	124.04 (32.56)	111.93 (35.42)	0.051
LVESV (mL)	52.49 (20.63)	54.59 (18.41)	50.87 (22.19)	0182
LVSV (mL)	65.00 (21.18)	69.47 (19.43)	61.55 (21.96)	0.013
LVEF (%)	57.13 (9.80)	56.98 (7.82)	57.24 (11.15)	0.884
LAAP (mm)	43.98 (4.40)	43.47 (4.14)	44.38 (4.57)	0.252
LAVI (mL/m^2^)	47.09 (12.28)	42.84 (9.43)	50.39 (13.24)	<0.001
LAEDVI (mL/m^2^)	34.22 (12.05)	29.15 (8.63)	38.14 (12.89)	<0.001
LAEF (%)	26.84 (10.09)	31.13 (7.99)	23.52 (10.35)	<0.001
RAA s (cm^2^)	22.29 (5.18)	21.30 (5.53)	23.02 (4.82)	0.071
RAA d (cm^2^)	16.66 (4.41)	16.07 (4.79)	17.09 (4.09)	0.061
s′ lat (cm/s)	6.57 (1.90)	7.13 (1.83)	6.13 (1.85)	0.002
e′ lat (cm/s)	11.57 (2.96)	12.37 (2.69)	10.95 (3.02)	0.007
s′ mid (cm/s)	5.46 (1.62)	6.18 (1.73)	4.91 (1.29)	<0.001
e′ mid (cm/s)	8.25 (2.33)	9.10 (2.63)	7.59 (1.82)	0.001
s′ mean (cm/s)	6.01 (1.66)	6.65 (1.68)	5.51 (1.46)	<0.001
e′ mean (cm/s)	9.91 (2.36)	10.74 (2.29)	9.27 (2.22)	<0.001
E (m/s)	0.88 (0.19)	0.83 (0.15)	0.93 (0.21)	<0.006
LAWMV (cm/s)	3.22 (1.07)	3.69 (0.84)	2.86 (1.09)	<0.001

Presented values are means ± standard deviations; d, diastolic; E, early filling wave; e′, early diastolic mitral annular velocity; IVS, intraventricular septum wall thickness; LAAP, left atrial anteroposterior diameter; LAEDVI, left atrial end-diastolic volume index; LAEF, left atrial emptying fraction; lat, measurement obtained from the lateral part of the mitral ring; LAWMV, left atrial wall motion velocity; LAVI, left atrial volume index; LVEDD, left ventricular end-diastolic diameter; LVEDV, left ventricular end-diastolic volume; LVEF, left ventricular ejection fraction; LVESD, left ventricular end-systolic diameter; LVESV, left ventricular end-systolic diameter; LVSV, left ventricular stroke volume; mean, averages of measurements taken from the medial and lateral parts of the mitral ring; mid, measurement obtained from the medial part of the mitral ring; RAA, right atrial area; RVOT, right ventricular outflow tract; s′ wave, systolic mitral annular velocity; s, systolic.

**Table 3:** Multivariate regression analysis including echocardiographic and clinical variables.

Variable	Univariate Analysis			Multivariable Analysis		
	OR	95% CI	*p*-Value	OR	95% CI	*p*-Value
LAVI (ml/m^2^)	0.94	0.91–0.98	0.001	0.96	0.92–1.00	0.050
e′ mean (cm/s)	1.36	1.13–1.63	0.001	1.18	0.96–1.47	0.116
LAWMV (cm/s)	2.29	1.54–3.39	<0.001	1.81	1.19–2.77	0.006
Male sex	2.74	1.29–5.83	0.009	1.86	0.79–4.39	0.156
Diuretics post	0.36	0.18–0.76	0.007	0.60	0.26–1.41	0.242

CI, confidence interval; e′ mean, early diastolic mitral annular velocity; LAWMV, left atrial wall motion velocity; LAVI, left atrial volume index; post, taken after cardioversion; OR, odds ratio.

**Table 4:** Multivariate regression analysis including echocardiographic variables.

Variable	Univariate Analysis			Multivariable Analysis		
	OR	95% CI	*p*-Value	OR	95% CI	*p*-Value
LAVI (ml/m^2^)	0.94	0.91–0.98	0.001	0.97	0.93–1.00	0.081
LAEF (%)	1.09	1.05–1.15	<0.001	1.04	0.99–1.10	0.082
e′ mean (cm/s)	1.36	1.13–1.63	0.001	1.19	0.95–1.49	0.132
E (m/s)	0.06	0.01–0.43	0.006	0.32	0.03–3.83	0.369
LAWMV (cm/s)	2.29	1.54–3.39	<0.001	1.72	1.10–2.69	0.017

CI, confidence interval; E, early filling wave; e′, early diastolic mitral annular velocity; LAEF, left atrial emptying fraction; LAWMV, left atrial wall motion velocity; LAVI, left atrial volume index. OR, odds ratio.

**Table 5:** The area under the curve analysis for LAWMV predicting sinus rhythm restoration after DCCV.

Variable	AUC (95% CI)	*p*-Value	Cut-Off Value (cm/s)	Sensitivity (%)	Specificity (%)	PPV (%)	NPV (%)
LAWMV	0.911 (0.862–0.961)	<0.001	3.0	100	81.3	100	18.7

AUC, area under the curve; LAWMV, left atrial wall movement velocity; NPV, negative predictive value; PPV, positive predictive value.

**Table 6:** The area under the curve analysis for LAWMV predicting sinus rhythm maintenance at 12 months after DCCV.

Variable	AUC (95% CI)	*p*-Value	Cut-Off Value (cm/s)	Sensitivity (%)	Specificity (%)	PPV (%)	NPV (%)
LAWMV	0.738 (0.651–0.825)	<0.001	3.0	0.927	0.493	0.586	0.897

AUC, area under the curve; LAWMV, left atrial wall movement velocity; NPV, negative predictive value; PPV, positive predictive value.

## Data Availability

The data underlying this article will be shared on reasonable request to the corresponding author.
